# RETRACTION: Combined Analysis of circRNA and mRNA Profiles and Interactions in Patients With Diabetic Foot and Diabetes Mellitus

**DOI:** 10.1111/iwj.70647

**Published:** 2025-04-23

## Abstract

**RETRACTION:** ZhaoW. , LiangJ. , ChenZ. , DiaoY. , and MiaoG. , “Combined Analysis of circRNA and mRNA Profiles and Interactions in Patients With Diabetic Foot and Diabetes Mellitus,” International Wound Journal 17, no. 5 (2020): 1183–1193, 10.1111/iwj.13420.32573975 PMC7949074

The above article, published online on 23 June 2020, in Wiley Online Library (http://onlinelibrary.wiley.com/), has been retracted by agreement between the journal Editor in Chief, Professor Keith Harding; and John Wiley & Sons, Ltd. Following an investigation by the publisher, all parties have concluded that this article was accepted solely on the basis of a compromised peer review process. The editors have therefore decided to retract the article. The authors did not respond to our notice regarding the retraction.

**RETRACTION:** W. Zhao , J. Liang , Z. Chen , Y. Diao , and G. Miao , “Combined Analysis of circRNA and mRNA Profiles and Interactions in Patients With Diabetic Foot and Diabetes Mellitus,” International Wound Journal 17, no. 5 (2020): 1183–1193, 10.1111/iwj.13420.32573975 PMC7949074

The above article, published online on 23 June 2020, in Wiley Online Library (http://onlinelibrary.wiley.com/), has been retracted by agreement between the journal Editor in Chief, Professor Keith Harding; and John Wiley & Sons, Ltd. Following an investigation by the publisher, all parties have concluded that this article was accepted solely on the basis of a compromised peer review process. The editors have therefore decided to retract the article. The authors did not respond to our notice regarding the retraction.

